# *Vibrio alginolyticus* influences quorum sensing-controlled phenotypes of acute hepatopancreatic necrosis disease-causing *Vibrio parahaemolyticus*

**DOI:** 10.7717/peerj.11567

**Published:** 2021-06-01

**Authors:** Panida Paopradit, Natta Tansila, Komwit Surachat, Pimonsri Mittraparp-arthorn

**Affiliations:** 1 Division of Biological Science, Faculty of Science, Prince of Songkla University, Hat Yai, Songkhla, Thailand; 2 Faculty of Medical Technology, Prince of Songkla University, Hat Yai, Songkhla, Thailand; 3 Division of Computational Science, Faculty of Science, Prince of Songkla University, Hat Yai, Songkhla, Thailand; 4 Molecular Evolution and Computational Biology Research Unit, Prince of Songkla University, Hat Yai, Songkhla, Thailand

**Keywords:** AHPND, Biofilms, Flagella, LuxR, Motility, OpaR, Probiotic, Quorum sensing, *V. alginolyticus*, *V. parahaemolyticus*

## Abstract

**Background:**

Acute hepatopancreatic necrosis syndrome (AHPND) caused by *Vibrio parahaemolyticus* strain (VP_AHPND_) impacts the shrimp industry worldwide. With the increasing problem of antibiotic abuse, studies on quorum sensing (QS) system and anti-QS compounds bring potential breakthroughs for disease prevention and treatment.

**Methods:**

In this study, the cell-free culture supernatant (CFCS) and its extract of *V. alginolyticus* BC25 were investigated for anti-QS activity against a reporter bacteria, *Chromobacterium violaceum* DMST46846. The effects of CFCS and/ or extract on motility, biofilm formation and extracellular polymeric substances (EPSs) of VP_AHPND_ PSU5591 were evaluated. Moreover, the effects of *V. alginolyticus* BC25 on virulence of VP_AHPND_ PSU5591 were investigated by shrimp challenge test. The potentially active anti-QS compounds presented in the extract and effect on gene expression of VP_AHPND_ PSU5591 were identified.

**Results:**

The CFCS of *V. alginolyticus* BC25 and its extract showed a significant anti-QS activity against the reporter bacteria as well as swimming and swarming motilities, biofilms, and EPSs production by VP_AHPND_ PSU5591. Transcriptome analysis revealed that *V. alginolyticus* BC25 extract significantly reduced the flagella genes involved in biofilm formation and iron-controlled virulence regulatory gene of VP_AHPND_ PSU5591. Whereas, the LuxR family transcriptional regulator gene, c-factor, a cell-cell signaling gene, and capsular polysaccharide were up-regulated. The potentially active anti-QS compounds identified in extract were Cyclo-(L-Leu-L-Pro), and Cyclo-(L-Phe-L-Pro). Furthermore, *V. alginolyticus* BC25 enhanced disease resistance against VP_AHPND_ PSU5591 in tested shrimp larvae.

**Conclusion:**

These findings suggest that *V. alginolyticus* BC25 could provide natural anti-QS and anti-biofilms compounds and has great ability to be used as biocontrol agent against VP_AHPND_ infection in shrimp aquaculture.

## Introduction

In recent years, the global shrimp aquaculture industry has suffered serious losses from acute hepatopancreatic necrotic disease (AHPND) outbreak caused by a unique *Vibrio parahaemolyticus* strain (VP_AHPND_). First mass mortality of shrimp caused by AHPND occurred in China in 2009. After that, this disease has spread to several countries including Vietnam, Malaysia, Thailand, Mexico, Philippines, and Latin America (FAO, 2013; Nunan et al., 2014; De la Pena et al., 2015). Almost 80% of the economic loss in shrimp industry was estimated in China due to the catastrophic disease (Lightner et al., 2013). Moreover, in Vietnam, the shrimp production was reduced from 70,000 tons in 2010 to 30,000 tons in 2012, respectively (Zorriehzahra & Banaedrakhshan, 2015). In Malaysia, AHPND decreased the shrimp production from 70,000 tons in 2010 to 40,000 tons in 2011 (Leano & Mohan, 2012). In Thailand, the shrimp production dropped from 600,000 tons in 2011 to less than 200,000 tons in 2014 (Thitamadee et al., 2016). Also in Mexico, the disease led to an economic loss of approximately 118 million USD (Schryver et al., 2014). To overcome the disease outbreak in shrimp aquaculture, several chemicals and antibiotics are used such as chlorine, potassium permanganate, ampicillin, streptomycin, sulfamethoxazole, fosfomycin and bicozamycin (Lai et al., 2015; Boonyawiwat et al., 2017). However, antibiotic resistance of VP_AHPND_ could potentially be transferred among bacterial species in aquaculture systems. It has been reported that VP_AHPND_ from penaeid shrimp in Mexico demonstrated a high level of resistance to tetracycline (≥5μg/mL) and carried the *tetB* gene coding for tetracycline resistance (Han et al., 2015). In the environment, multiple bacterial species coexist as communities. Bacteria have a mechanism for cell to cell communication as a quorum sensing system (QS) through the use of small signaling molecule called autoinducer. They use signaling molecule to coordinate their behavior such as bioluminescence, biofilm formation, spore formation, motility and developing antibiotic resistance (Ryan & Dow, 2008). QS system in *V. parahaemolyticus* is similar with that of *V. harveyi* (Zhang et al., 2012). *V. parahaemolyticus* produces harveyi autoinducer 1 (HAI-1), autoinducer 2 (AI-2) and cholerae autoinducer 1 (CAI-1). At low cell density, a phosphate group from the receptor transferred to LuxU and LuxO regulator. The phosphorylated LuxO activates the small quorum regulatory RNAs (Qrrs) transcription and promotes the translation of AphA master regulator and inhibit the mRNA of LuxR or OpaR regulator. At high cell density, the autoinducers are bound to receptors lead to dephosphorylation of LuxU and LuxO regulator. The Qrr genes are not transcribed, resulting in an expressed LuxR or OpaR regulator (Gode-Potratz & McCarter, 2011; Zhang et al., 2012). It has been reported that OpaR regulator regulates colony morphology, motility, capsule polysaccharide (CPS) production, type III and VI secretion system production (T3SS, T6SS) (Gode-Potratz & McCarter, 2011; Zhang et al., 2012; Wang et al., 2013; Salomon, Klimko & Orth, 2014; Kernell Burke et al., 2015; Wu et al., 2019). Therefore, QS is one of the alternative strategies for controlling virulence and behavior of bacteria.

*V. alginolyticus* is closely related to *V. parahaemolyticus* due to their similar biochemical characteristics and flagellar systems (Kawagishi et al., 1995; Montieri et al., 2010). In 1965, it has been reported from a taxonomic numerical study that *V. alginolyticus* is regarded as biotype 2 *V. parahaemolyticus*. *Vibrio parahaemoltyicus* historically had included two biotypes. The similarity value between two biotypes was approximately 80%, but the differentiating characters was sucrose fermentation, Voges-Proskauer reaction and growth in the peptone water containing 10% NaCl. Thus, the second biotype be moved and put into a new species to which the name *Vibrio alginolyticus* (Zen-Yoji et al., 1973). In addition, *V. alginolyticus* has long been employed as a probiotic in various shrimp hatcheries. It can be isolated from shrimp culture water and reportedly associates with healthy larvae and juvenile shrimp (Rengpipat et al., 2000). It is also found in the hepatopancreas and intestine of healthy *Penaeus monodon* and *Litopenaeus vannamei* (Gomez-Gil et al., 1998; Vandenberghe et al., 1999). *V. alginolyticus* effectively stimulates the shrimp immune response (Gullian, Thompson & Rodrýguez, 2004). In addition, it has been reported that *V. alginolyticus* UTM102 isolated from the gastrointestinal tract of adult shrimp *L*. *vannamei* showed antagonism against shrimp-pathogenic *V. parahaemolyticus* PS-017 (Balcázar, Rojas-Luna & Cunningham, 2007). For this reason, the aims of this study were to analyze the efficacy of *V. alginolyticus* BC25 isolated from the microbiota of marine mollusk for its anti-quorum sensing controlled phenotypes of VP_AHPND_ and ability to enhance disease resistance against this shrimp pathogen. The active fractions of *V. alginolyticus* BC25 extract were identified and effects on gene expression of VP_AHPND_ PSU5591 were also studied.

## Materials & Methods

### Bacterial strains and culture conditions

The natural marine bacterium *V. alginolyticus* BC25 was isolated from the microbiota of marine blood cockle collected in Songkhla province, Thailand and is deposited in the Thailand Bioresource Research Center (TBRC), Pathum Thani, Thailand with the Accession Numbers TBRC12771. VP_AHPND_ PSU5591 was isolated from the hepatopancreas of VP_AHPND_-infected shrimp in Songkla, Southern Thailand. *Chromobacterium violaceum* DMST46846 used in this study was purchase from DMST Culture Collection, Department of Medical Sciences Thailand. Bacteria were cultured at 30 °C in Luria-Bertani (LB) or LB containing 1% NaCl medium.

### Screening for anti-QS activity

The cell-free culture supernatant (CFCS) of *V. alginolyticus* was screened for its anti-QS activity by testing with *C. violaceum*. To prepare the CFCS of *V. alginolyticus*, the bacteria were cultured in LB broth with 1% NaCl, and incubated at 30 °C for 24 h. Cells were removed by centrifugation at 12,000 × G for 15 min. The CFCS was sterilized by sonication. Briefly, CFCS was placed in 50 mL sterile conical and sonicated by submerging the probe tip of a 20 kHz (size 12.7 mm), 45% amplitude with pulse mode 15 s and a 5 s rest for 15 min (Joyce, Al-Hashimi & Mason, 2011). The inhibition of violacein production in *C*. *violaceum* was analyzed as described previously with slight modification (Fitriani, Ayuningtyas & Kusnadi, 2016). In brief, *C*. *violaceum* was cultured in LB broth, incubated at 30 °C for 4 h, and adjusted to 0.5 McFarland (1.0 × 10^8^ CFU/mL). Before treatment, 30 µL of freshly grown *C*. *violaceum* culture was mixed with different concentrations (6.25, 25, 50, and 100%, v/v) of CFCS and incubated at 30 °C with shaking 150 rpm for 24 h. The total of viable bacteria was counted on LB agar. All treatments were centrifuged at 12,000 × G for 10 min and the pellets *C*. *violaceum* were resuspended in two mL dimethylsulfoxide (DMSO) to extract the violacein. Extracts were centrifuged at 12,000 × G for 10 min and 200 µL of CFCS was read at 585 nm using a microplate reader (LUMIstar Omega, BMG LABTECH, Germany) to check for the presence (or absence) of violacein.

### Preparation of *Vibrio alginolyticus* extract and anti-QS assay

*V. alginolyticus* was cultured for 24 h at 30 °C in LB broth with 1% NaCl. The culture was centrifuged for 15 min at 12,000 × G. The sterilized CFCS was extracted using an equal volume of ethyl acetate (CH_3_COOCH_2_CH_3_), concentrated using a rotary evaporator at 45 °C, dissolved in DMSO, and stored at −20 °C (Nithya, Begum & Pandian, 2010c). The anti-QS activity of different concentration of *V. alginolyticus* extract (25, 50, and 100%, v/v) was re-tested by an agar well assay using *C*. *violaceum* as a reporter strain. *Serratia marcescens* and DMSO were considered as positive and negative control, respectively. After 24 h of incubation, the transparent halo of violacein inhibition indicates anti-QS activity.

### Effect of *V. alginolyticus* on QS-controlled phenotypes of VP_AHPND_

#### Swimming and swarming motility assay

Motility tests was performed as described previously with slight modifications (Zhang et al., 2016). These assays were conducted on LB agar with 1% NaCl supplemented with or without different concentrations of CFCS (40, 60, and 80%) and its extract. For swimming motility test, VP_AHPND_ was cultured in LB broth, and 3 µL aliquots (0.5 McFarland) was spotted on the center of 0.3% (w/v) agar plates. The diameter zone of the motility were measured and photographed. For swarming motility test, VP_AHPND_ was streaked as a straight line on 1.5% (w/v) agar and incubated at 30 °C for 18 h. The diffuse zone surrounding the inoculum lines were measured and photographed.

### Biofilm formation assay

The anti-biofilm activity assay of VP_AHPND_ was determined as described previously with slight modifications (Nesper et al., 2001). Briefly, an overnight VP_AHPND_ culture in LB broth was added to a 96-well microtiter plate with or without *V. alginolyticus* extract (2.5, 5, and 10%) and incubated at 30 °C for 24 h. Planktonic cells were removed by washing the wells with sterile water, fixed with 2.5% glutaraldehyde, and stained with 0.4% (w/v) crystal violet. After that ethanol-acetone (80:20, v/v) was added. The OD was determined at 570 nm in a microplate reader (LUMIstar Omega, BMG LABTECH, Germany).

The effect of *V. alginolyticus* extract on EPSs in biofilm was visualized by scanning electron microscopy (SEM) as described previously with minor modifications (Singh, Mishra & Jha, 2017). Briefly, biofilms of VP_AHPND_ grown on glass coverslips (1.0 × 1.0 cm) submerged in LB broth with or without *V. alginolyticus* extract in 24-well plate were gently washed with phosphate buffer saline (PBS) to remove planktonic cells. Afterwards the cells were fixed with 2.5% glutaraldehyde (v/v) at 4 °C for 12 h. After dehydrating in a gradient ethanol series (30–100%, v/v) for 15 min each, the dried samples were coated with gold and investigated under a SEM (Quanta 400, Thermo Fisher Scientific, USA).

### Gene expression analysis

The Effect of *V. alginolyticus* extract on gene expression of VP_AHPND_ was analyzed using RNA sequencing (RNA-seq) analysis. VP_AHPND_ was treated with or without *V. alginolyticus* extract as a control at 30 °C for 24 h. The bacterial culture was centrifuged at 8,000 × G for 10 min, and RNA extraction was initially done by stabilizing RNA using RNAprotect Bacteria Reagent (Qiagen, Germany). Total RNA from the two samples was extracted using the TRIzol™ Reagent (Invitrogen; Thermo Fisher Scientific, Inc., Waltham, MA, USA) following the manufacturer’s protocol. All RNAs were stored at −80 °C immediately until use. RNA sequencing was performed using a NovaSeq 6000 system (Macrogen, Korea). Sequence data was mapped to reference genome with Bowtie software 1.1.2 (Bolger, Lohse & Usadel, 2014). Read count per gene was extracted from known gene annotations with HTSeq program version 0.10.0 (Anders, Theoder Pyl & Huber, 2015). The evaluating gene expression profiles were performed using FPKM method (fragments per kilobase of transcript per million mapped reads) as a normalization value. Statistical analysis was performed using fold change, exactTest using edgeR per comparion pair (Robinson, McCarthy & Smyth, 2010). The significant results are selected on conditions of lfcl ≥ 2 and exactTest raw *p*-value < 0.05

### Separation and identification of *V. alginolyticus* extract

*V. alginolyticus* extract was fractionated by high-performance liquid chromatography (HPLC) 1260 (Agilent Technologies, USA). One hundred microliter of extract was injected onto a non-polar reverse-phase C18 column (50 × 2.1 mm, Waters, CA, USA). The mobile phase was methanol and water (20:80, v/v) at 30 °C at a flow rate of 0.2 mL/min. Each fraction was collected, concentrated by rotary evaporator, and re-tested for anti-QS activity as described above. The positive fractions were further analyzed by gas chromatograph-mass spectrometer (GC-MS) (Agilent Technologies 5977A, USA) as described previously (Savka et al., 2015). The spectra were matching with the National Institute of Standards and Technology (NIST) reference library to identify the anti-QS compounds based on retention indices. The molecular structure of active compound was confirmed by nuclear magnetic resonance spectroscopy (NMR). The active compound was dissolved in deuterated MeOH (CD_3_OD), and subjected to ^1^H and ^13^C NMR analyses using a NMR Bruker/Avance (500 MHz for ^1^H and 125MHz for ^13^C).

### *V. alginolyticus* treatment and challenge test

*Litopenaeus vannamei* were obtained from a shrimp farm in Songkhla province, Thailand. The shrimp larvae (postlarval age = 25 days) were acclimatized for 3 day before transferring to glass tanks containing 3 L artificial sea water (Marinum, Thailand) (15 shrimp/tank). The experiment consisted of four groups: group 1 (treated with *V. alginolyticus* at a concentration of 10^5^ CFU/mL to investigate the possible virulence of *V. alginolyticus*); group 2 (treated with VP_AHPND_ at a concentration of 10^6^ CFU/mL as positive control) and group 3 (treated with *V. alginolyticus* three days before infection with VP_AHPND_ to test the ability of *V. alginolyticus* for the control of VP_AHPND_ disease in shrimp) and only shrimp larvae (no any treatment) as negative control , as described previously (Rodriguez et al., 2011; Joshi et al., 2014). Each treatment was carried out in triplicates. Mortality was recorded until 72 h post infection.

### Statistical analysis

Effects of different treatments were tested by one-way ANOVA analyses using the SPSS software version 14 (SPSS Inc., USA). Differences supported by a *p*-value of less than 0.05 were considered as significant.

## Results

### *V. alginolyticus* showed anti-QS activity by inhibiting violacein production

**Figure 1:**
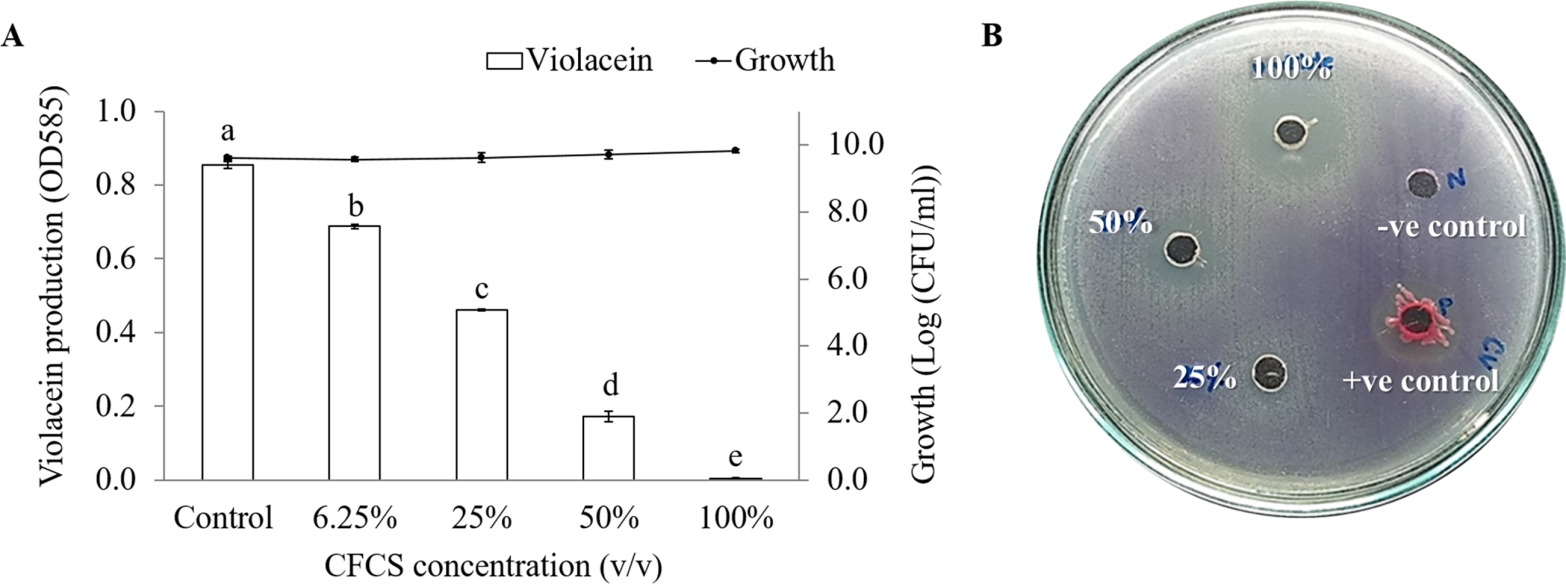
The inhibition of violacein production in *C. violaceum* DMST46846. (A) Different concentration of CFCS of *V. alginolyticus* BC25 was used to detect the reduction of purple pigment as indicator for QS activity. Different letters (a, b, c, d, and e) are significant difference (*p* < 0.001) among the treatments. (B) Screening of anti-QS activity of *V. alginolyticus* BC25 extract by agar well diffusion method. DMSO solvent and *S. marcescens* were used as negative and positive control, respectively.

A lack of the purple pigment violacein from indicator strain indicates a potential anti-QS result. Our results revealed that CFCS of *V. alginolyticus* and its extract showed anti-QS activity. The reduction of violacein production in *C. violaceum* are CFCS concentration-dependent manner. About 79.7% inhibition (*P* < 0.001) was observed with the addition 50% (v/v) CFCS and without affecting to *C. violaceum* growth (Fig. 1A). Different concentrations of *V. alginolyticus* extract on violacein production were re-tested by agar well diffusion assay. As expected, the violacein production reduced with the increasing concentration of *V. alginolyticus* extract. However, *V. alginolyticus* extract inhibited the growth of *C. violaceum* at high concentration (Fig. 1B).

### *V. alginolyticus* inhibited motilities and biofilms of VP_AHPND_

**Figure 2:**
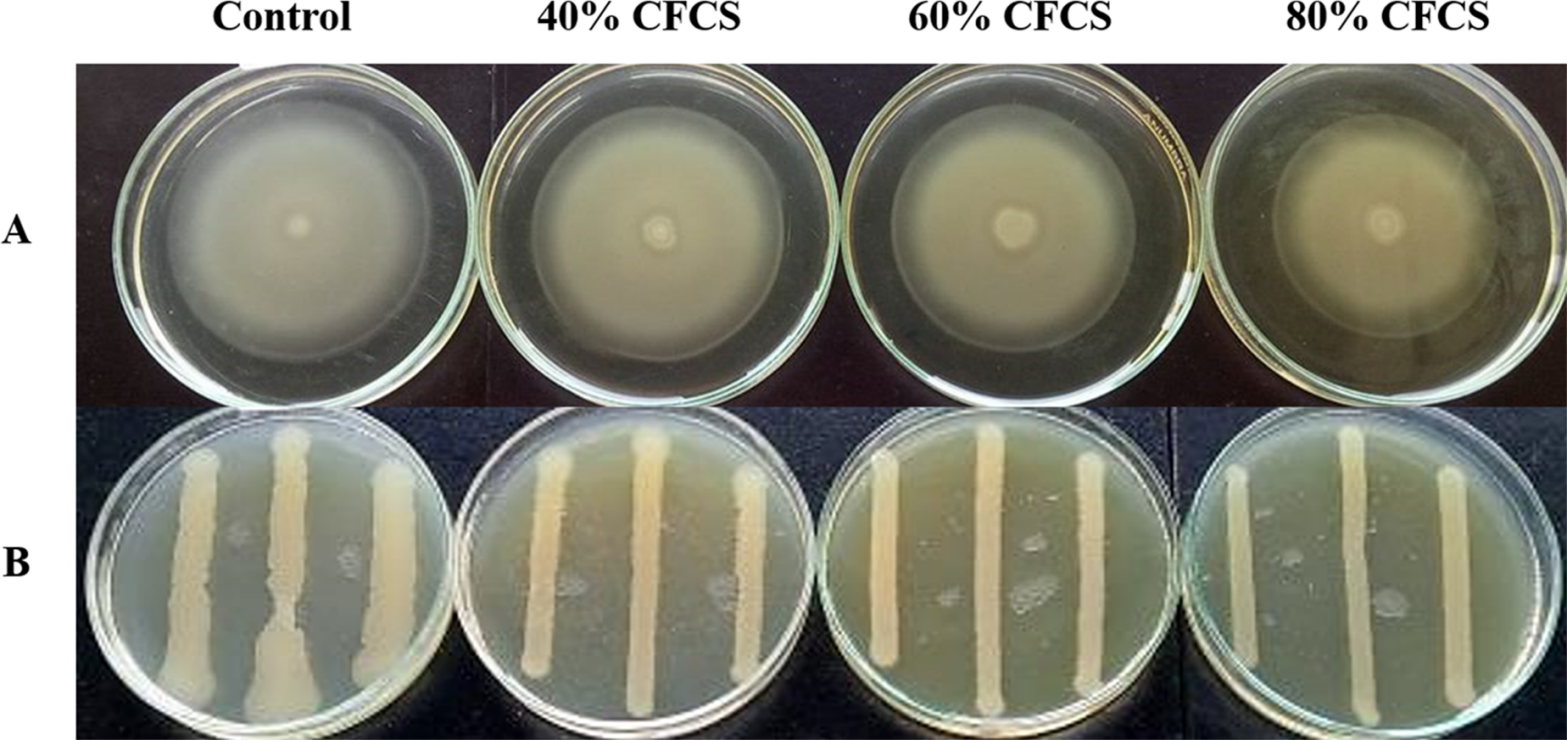
Motility of VP_AHPND_ PSU5591 in different concentration of CFCS of *V. alginolyticus* BC25. (A) Swimming motility of VP_AHPND_ on 0.3% (w/v) LB agar by spot method. (B) Swarming motility of VP_AHPND_ on 1.5% (w/v) LB agar by streak method. The motility zone was measured and provided as mean ± standard deviation as described in Table 1.

**Figure 3:**
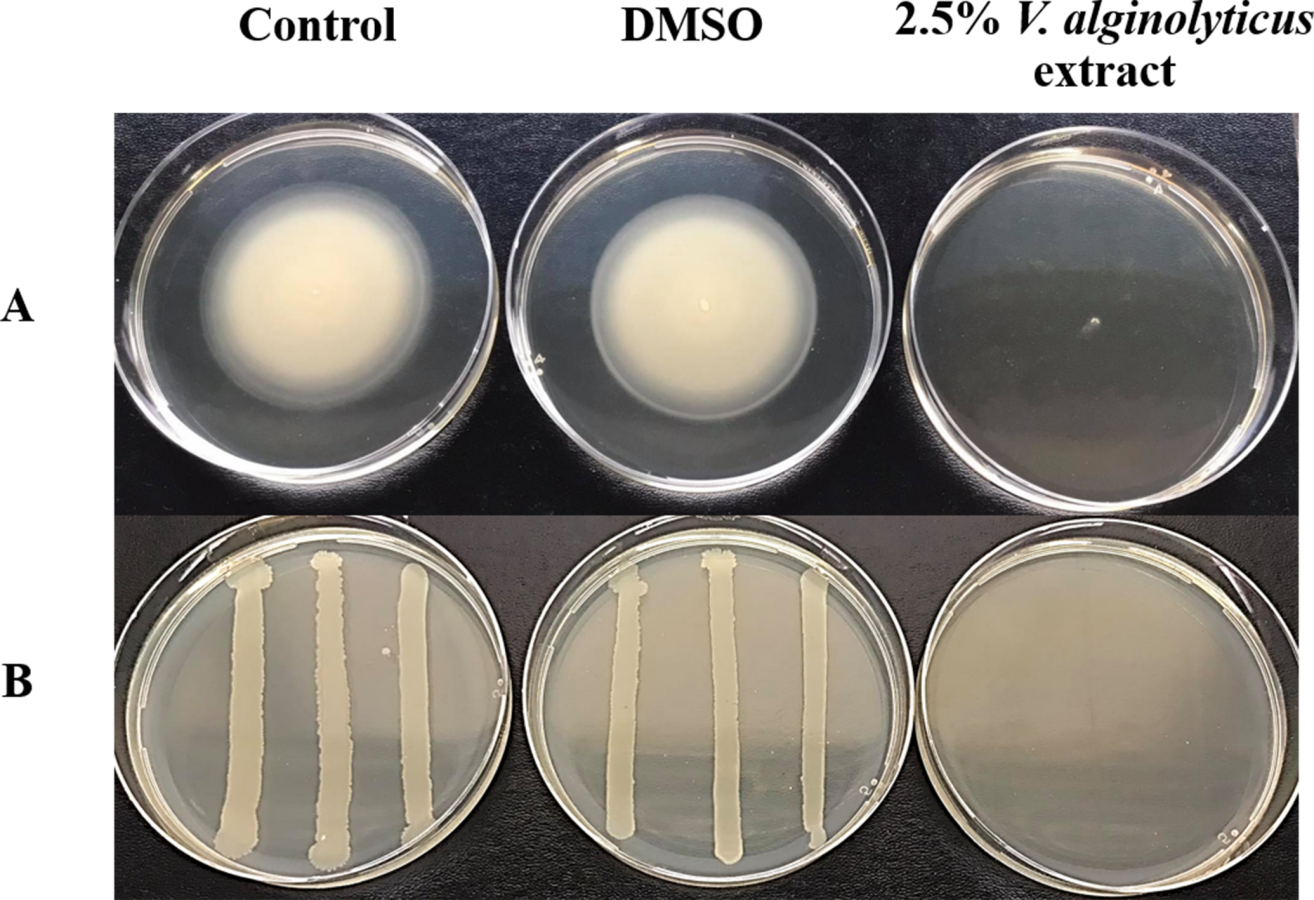
Motility of VP_AHPND_ PSU5591 in the presence of *V. alginolyticus* extract. (A) Swimming motility of VP_AHPND_ on 0.3% (w/v) LB agar by spot method. (B) Swarming motility of VP_AHPND_ on 1.5% (w/v) LB agar by streak method. The motility zone was measured and provided as mean ± standard deviation as described in Table 2.

**Figure 4:**
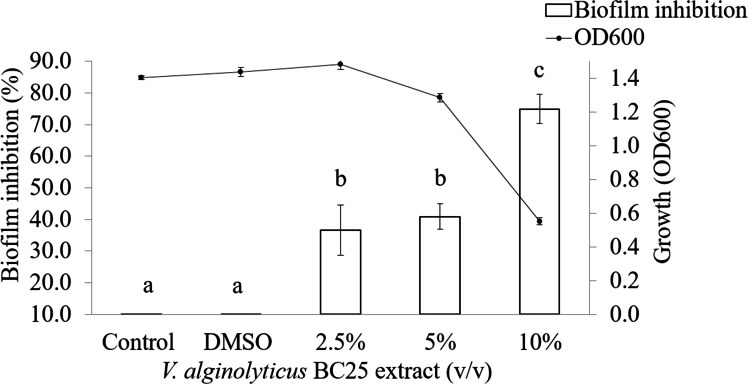
Inhibition of biofilm formation in VP_AHPND_ PSU5591 by different concentration of the *V. alginolyticus* BC25 extract. Different letters (a, b, and c) are significant difference (*p* < 0.001) among the treatments.

**Figure 5:**
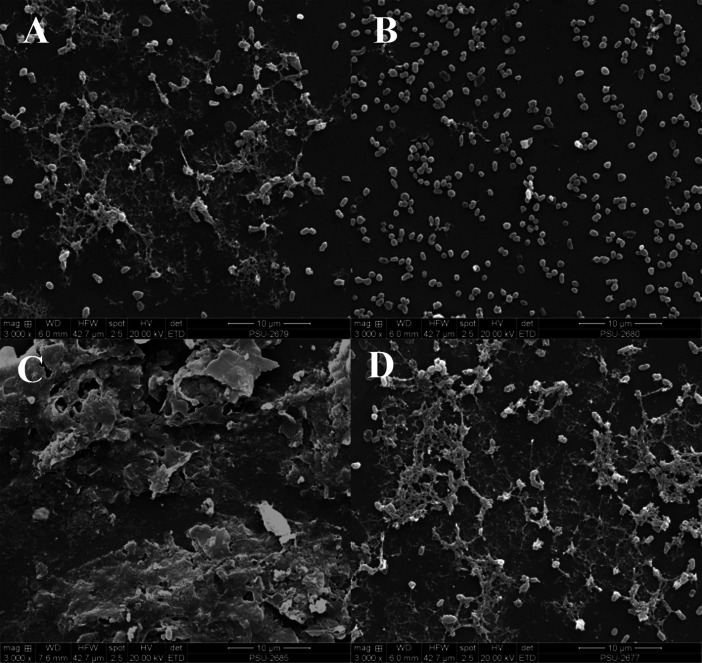
Inhibition of EPSs in biofilm of VP_AHPND_ PSU5591 by 2.5% (v/v) *V. alginolyticus* BC25 extract in 24 and 48 h cultivation. Representative photographs by SEM. (A) control at 24 h, (B) treated at 24 h, (C) control at 48 h and (D) treated at 48 h.

It was observed that CFCS of *V. alginolyticus* reduced the swimming and swarming motilities of VP_AHPND_ (Fig. 2). The CFCS at 80% significantly decreased the diameter of the swimming and at 40 to 80% CFCS also decreased the swarming area, which significantly differs (*p* < 0.01) when compared with control as shown in Table 1. As expected that *V. alginolyticus* extract greatly reduced the swimming and swarming motilities of VP_AHPND_ at 2.5% (v/v) with significant differences (*P* < 0.001) (Fig. 3, Table 2). The anti-biofilm activity of *V. alginolyticus* extract with a different concentration (2.5, 5, and 10%) was tested against VP_AHPND_. About 36.6% inhibition of biofilm formation with *P* <0.001 was noticed with 2.5% (v/v) extract without significant effect on cell growth. However, the growth inhibition was observed with high extract concentrations used (Fig. 4). Thus, similar concentration of *V. alginolyticus* extract was employed to test for a possible effect on the architecture of the VP_AHPND_ biofilm by SEM analysis. A thick and dense biofilm along with EPSs production was observed in control after 24 and 48 h incubation (Figs. 5A, 5C), whereas the reduction in VP_AHPND_ EPSs was observed in treated samples (Figs. 5B, 5D).

**Table 1:** The swimming and swarming zone diameter of VP_**AHPND**_ PSU5591 in the presence of different concentrations of CFCS of *V. alginolyticus* BC25. Data are provided as mean ± standard deviation of triplicate, Different letters (a, and b) are significant difference (*p* < 0.01) among the treatments.

Motility	CFCS concentration (%, v/v)	Motility zone (mm)
Swimming	Control	66.20 ± 0.21^a^
	40	64.70 ± 0.04^a^
	60	61.78 ± 0.41^a^
	80	55.28 ± 0.65^b^
Swarming	Control	10.68 ± 1.08^a^
	40	7.12 ± 0.42^b^
	60	6.87 ± 0.46^b^
	80	6.12 ± 0.40^b^


**Table 2:** The swimming and swarming zone diameter of VP_AHPND_ PSU5591 in the presence of *V*. *alginolyticus* extract. Data are provided as mean ± standard deviation of triplicate, Different letters (a, and b) are significant difference (*p* <0.001) among the treatments.

Motility	Concentration (%, v/v)	Motility zone (mm)
Swimming	Control	46.52 ± 1.32^b^
	DMSO	51.10 ± 0.92^b^
	2.5% extract	4.60 ± 0.41^a^
Swarming	Control	7.15 ± 0.69^b^
	DMSO	5.38 ± 0.12^b^
	2.5% extract	0.97 ± 0.16^a^

### *V. alginolyticus* extract affected the expression of QS regulatory genes of VP_AHPND_

**Figure 6:**
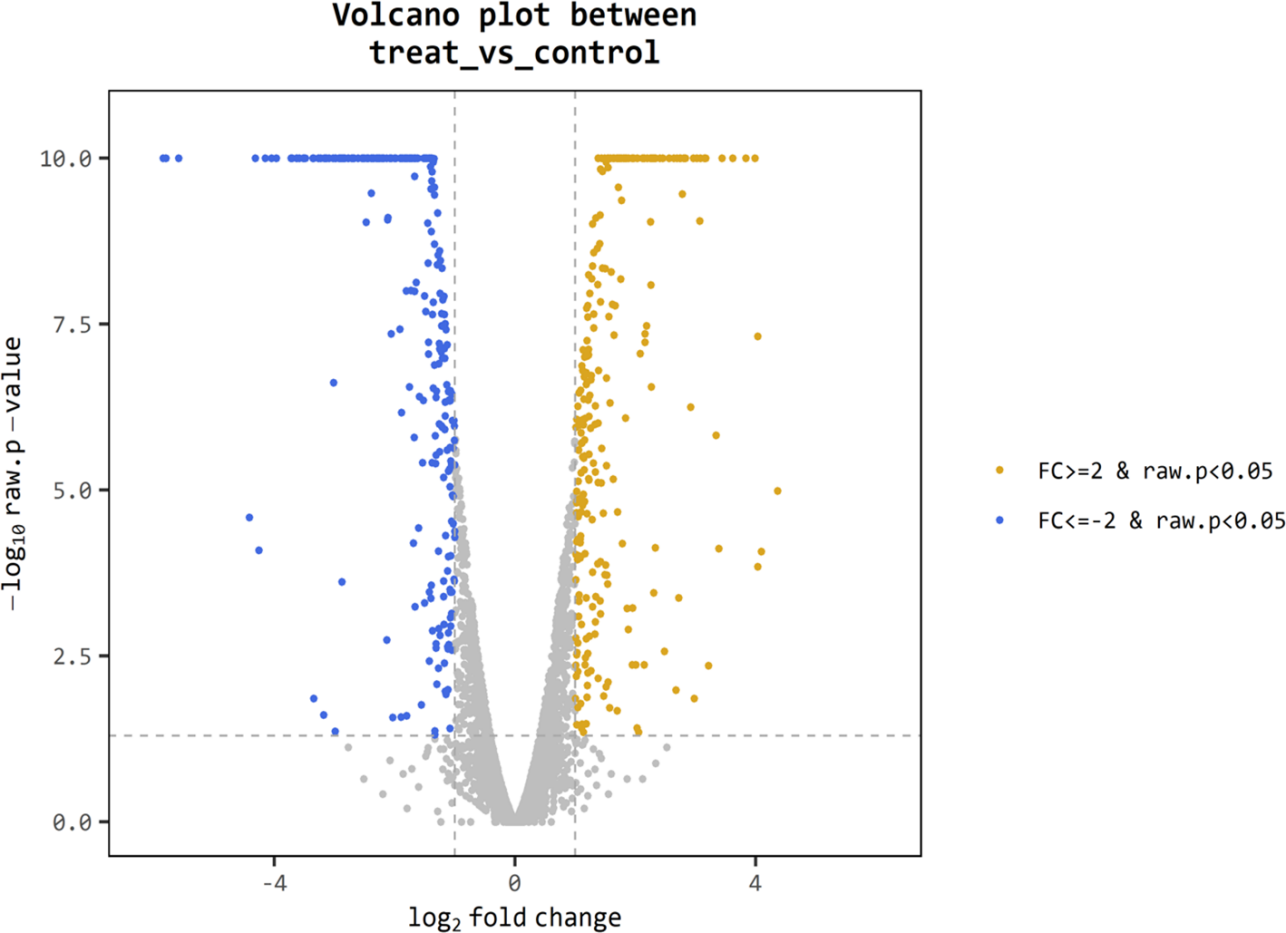
Differentially expressed genes (DEGs) of VP_AHPND_ PSU5591 by volcano plot. In total, 524 genes indicate different expression. Blue shows down-regulated expression, brown shows up-regulated expression and gray shows no significant difference when comparing control and treat sample.

**Figure 7:**
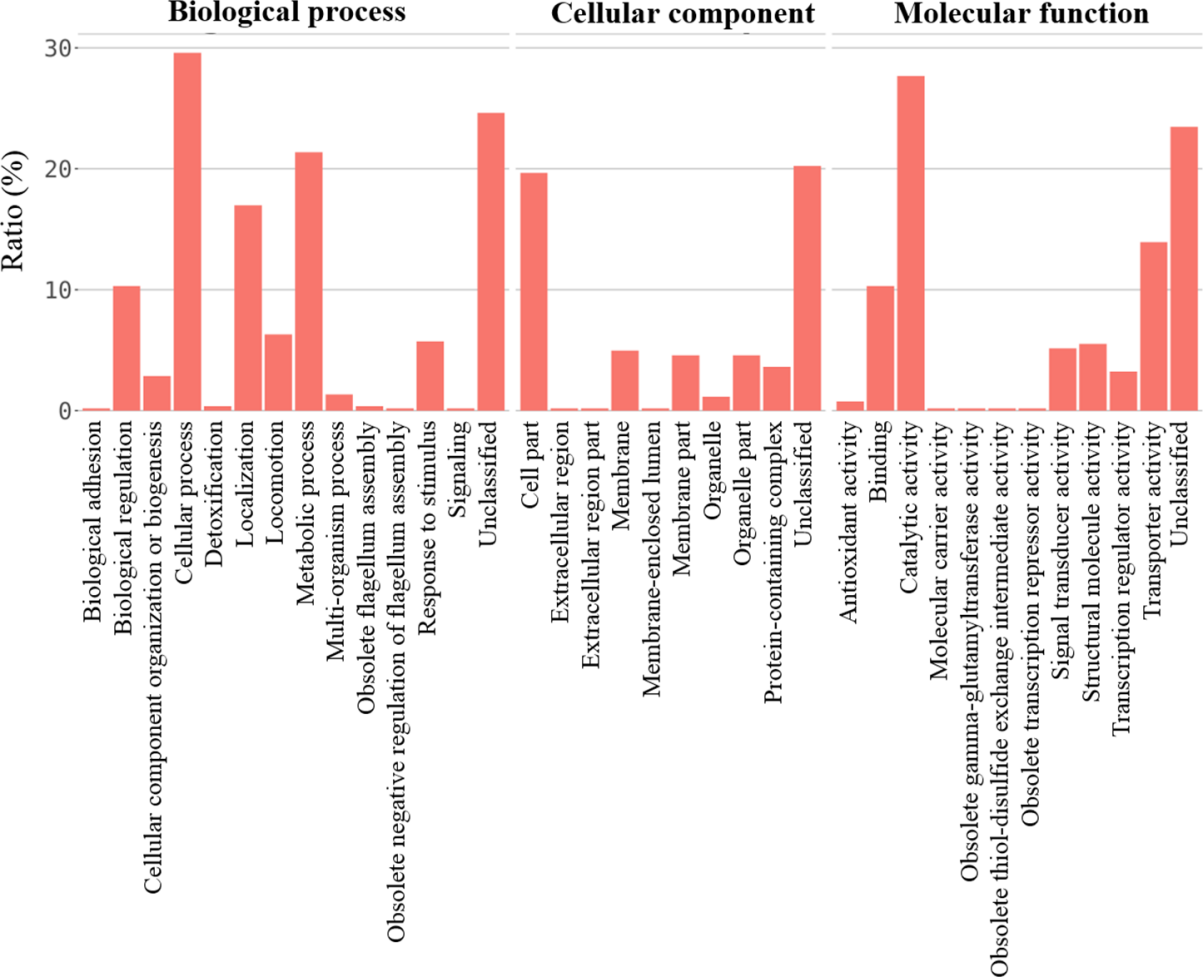
The gene expression associated to functional categories of VP_AHPND_ PSU5591 culture in the presence of *V. alginolyticus* BC25 extract.

For better understanding of the effect of *V. alginolyticus* extract on global gene expression of VP_AHPND_, cultures grown with or without 2.5% (v/v) extract were analyzed using RNA-Seq. A total of 6.3 Gb sequence data, including 62,669,812 raw reads and 48,830,934 mapped reads were obtained. The sequencing raw reads were deposited in the Sequence Read Archive (SRA) of the National Center for Biotechnology Information (NCBI) with GCA_000196095.1 reference genome and accession number SRR12398138, SRR12459902. The Q20 and Q30 percentages were higher than 98% and 95%, respectively (Table 3). A *p*-value less than 0.05 were chosen as limitation for screening of differentially expressed genes (DEGs). The RNA-seq data showed that 524 genes were differentially expressed, of which 259 were up-regulated and 265 were down-regulated. The volcano plot of DEGs showed the genes affected by *V. alginolyticus* extract were either down-regulated (blue dots, 265 genes) or up-regulated (yellow dots, 259 genes) (Fig. 6). Of the DEGs, three domains including biological process (14 subcategories): cellular process, metabolic process, localization, and biological regulation; cellular component (10 subcategories): cell part, membrane part, and organelle part; and molecular function (12 subcategories): catalytic activity, transporter activity, binding, signal transducer activity, and structural molecule activity were identified (Fig. 7). Interestingly, the expression of motility genes were highly affected by *V. alginolyticus* extract (Table S1).

**Table 3:** Summary of the RNA sequencing data.

Sample id	Total reads^a^	Mapped reads^b^	GC (%)^c^	Q20 (%)^d^	Q30 (%)^e^
control	32,451,134	26,471,918	49.03	98.96	96.17
treat	29,618,810	22,359,016	49.31	98.89	95.98

**Notes.**

a Total reads: Total number of reads.

b Mapped reads: Number of reads mapped to reference.

c GC (%): GC content.

d Q20 (%): Ratio of bases (phred quality score greater than or equal to 20).

e Q30 (%): Ratio of bases (phred quality score greater than or equal to 30).

### Separation and identification of *V. alginolyticus* extract

A total of seven fractions of *V. alginolyticus* extract were collected and individually screened for anti-QS activity using a reporter strain, *C*. *violaceum* by plate assay. The zone of QS inhibition was observed in four fractions collected (no. 1, 4, 5, and 7; Fig. S1). Therefore, these fractions were selected for further characterization by gas chromatography-mass spectrometry (GC-MS) analysis. The chromatogram showed a predominant peak at the retention time 19.44 min for fraction 1, 20.62 min for fraction 4, 27.36 min for fraction 5, and 27.38 min for fraction 7 (Dataset S1), respectively. A major mass spectral peak detected at m/z 154 for fraction 1 and 4, as well as at m/z 125 for fraction 5 and 7, was considered as active fraction. The detected mass spectra indicated some resemblance to Cyclo-(L-Leu-L-Pro) for fraction peak 1, 4 and Cyclo-(L-Phe-L-Pro) for fraction peak 5, 7 in the GC-MS library. The ^1^H NMR spectrum (500 MHz, MeOH, *J* in Hz) indicated the presence of 18 hydrogen atoms with value *δ* 0.92−0.95 (3H, 0.93 (*d*, *J* = 7.0 Hz)), *δ* 1.07−1.12 (3H, 1.09 (*d*, *J* = 7.0 Hz)), *δ* 1.78−1.80 (2H, m), *δ* 1.46 (1H, m), *δ* 1.83−1.98 (4H, 1.84 (*d*, *J* = 1.50 Hz), 1.94 (m), 1.95 (*d*, *J* = 1.50 Hz), 1.85 (m)), *δ* 4.58 (1H, (*d*, *J* = 25.0 Hz), *δ* 4.86 (1H, *s*), *δ* 3.47−3.61 (2H, 3.50 (m), 3.59 (m)), *δ* 6.69 (1H, *s*). The ^13^C NMR spectrum (125 MHz, MeOH) suggested the presence of 11 carbon atoms thus, confirming that the active compound was Cyclo-(L-Leu-L-Pro). The ^13^C NMR spectrum showed methane carbon in aliphatic region at *δ* 59.08 and 53.76 ppm. Two quaternary carbons of two C=O groups at *δ* 165.9 and 169.3 ppm were observed. Two methyl carbons were showed at *δ* 22.23 and 22.10 ppm. Furthermore, four methylene and a third methine carbons displayed at *δ* 47.08, 45.03, 28.48, 22.35, and 22.50 ppm, respectively. The NMR spectrum were compared with a standard compound in NMR Predict (http://www.nmrdb.org) and found to be similar to previous reported for Cyclo-(L-Leu-L-Pro) (Lalithal et al., 2016; Putra & Karim, 2019). Representative ^1^H-NMR, ^13^C-NMR spectra and 2D NMR (HMBC, HSQC) as shown in the Fig. S2.

### *V. alginolyticus* treatment and challenge test against VP_AHPND_

**Figure 8:**
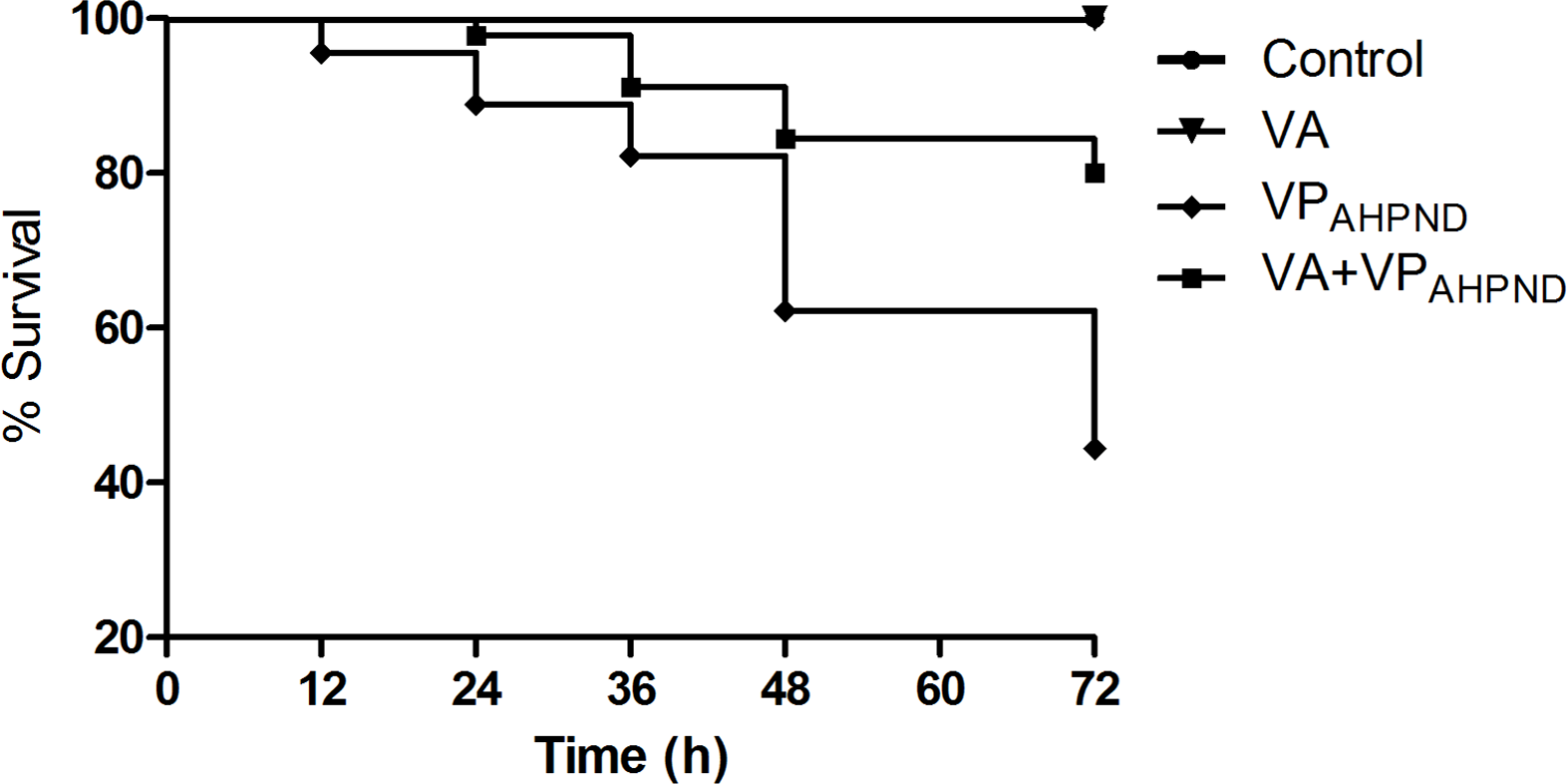
Kaplan–Meier survival of *Litopenaeus vannamei* shrimp infection assay.

In untreated group infected with VP_AHPND_ alone, the mortality of shrimp started at 12 h post infection, and reached 55.56% mortality within 72 h. However, with shrimp treated with *V. alginolyticus* (10^5^ CFU/mL) prior to infection with VP_AHPND_, the mortality of shrimp started at 24 h post infection and 80% survival was observed 72 h post-infection. During 72 h of the study, 100% survival was observed in shrimp larvae treated with *V. alginolyticus* suspensions (10^5^ CFU/mL) (Fig. 8).

## Discussion

Many antibiotics-resistant bacteria have occurred worldwide due to long-term use of antibiotics. There is an urgent need to develop alternative bacterial control strategies. QS inhibition has been proposed as a new strategy to control bacterial infection by interfering the expression of virulence factors and marine organisms have been proposed as an important source for the discovery of novel anti-QS compounds (Dobretsov, Dahms & Qian, 2006). Thus, this study evaluates the anti-QS activity of *V. alginolyticus* BC25 on reporter bacteria and investigates its effect on phenotypes of VP_AHPND_ PSU5591.

*C. violaceum* was used for the preliminary screening of marine bacterium with anti-QS activity. This bacteria produce the purple pigment violacein except its QS system is obstructed (Fitriani, Ayuningtyas & Kusnadi, 2016). QS system of *C. violaceum* consists of two regulatory protein CviI and CviR that are homologus to LuxI and LuxR systems, respectively. The violacein production is regulated by *vio* operon (*vioA*, *vioB*, *vioC*, *vioD*, and *vioE*) through a QS system mediated by AHL (August et al., 2000). Findings from the present study revealed that CFCS of *V. alginolyticus* (6.25 to 50%) could have influenced QS system of *C. violaceum.* About 79.7% inhibition in the violacein production was detected with 50% (v/v) CFCS and without affecting to cell growth. It is possible that the addition of *V. alginolyticus* CFCS affected the transcription of *vio* gene (encoding violacein production). Similar research revealed that the cell pellet and supernatant extract of *V. alginolyticus* strain M3-10 at concentration 0.8 mg/mL reduced the violacein production without affecting the cell growth of *C. violaceum* ATCC 12472 and was therefore selected for QS inhibition assay against *Pseudomonas aeruginosa* (Reina et al., 2019).

In this study, global RNA-seq was used to explore the possible effects of *V. alginolyticus* extract on VP_AHPND_. The RNA-seq data showed the differential expression of 524 genes, of which 259 were up-regulated and 265 were down-regulated. Most of these genes are involved in quorum sensing, motility, virulence, and transport. Flagella-based motility is an important mode of locomotion of bacteria. In *V. parahaemolyticus*, it has two kinds of flagella include polar flagella (*fla* gene system) and lateral flagella (*laf* gene system) (Kojima et al., 2011). Polar flagellum is responsible for bacterial swimming in liquid, while lateral flagellum is required for bacterial swarming in solid surface (McCarter, 2004; Jaques & McCarter, 2006). In the present study, we examined the effect of CFCS of *V. alginolyticus* on swimming and swarming of VP_AHPND_ by the spot method. However, swarming zone of VP_AHPND_remains unclear (data not shown). For this reason, the streak method used for the swarming test. We demonstrated that at 40 to 80% CFCS of *V. alginolyticus* decreased the swimming motility and also influenced the swarming motility of VP_AHPND_ in the plate assay. As expected, a majority of flagellar genes encoded for the flagellar structure and flagellin were down-regulated in the presence of *V. alginolyticus* extract. Thus, it is possible that the decreased swimming of VP_AHPND_ is caused by the regulation of *PomA* and *flaABCFGI* genes, which control the function of polar flagellar. Also, *flgABCDEFGKLMN*, *fliADMS* and *flhFG* genes are associated with the function of lateral flagellar.

**Figure 9:**
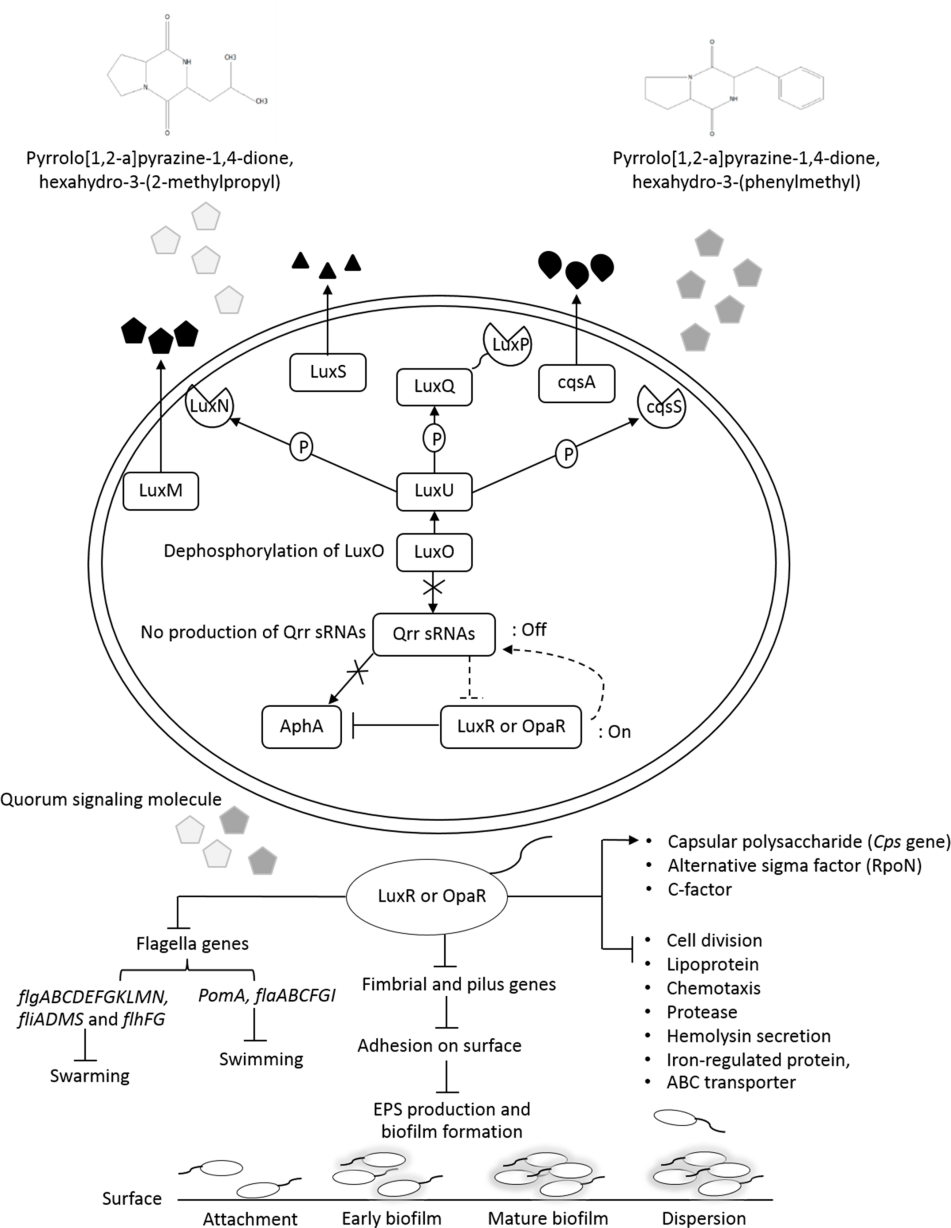
The transcriptional regulation model of QSI in VP_AHPND_ PSU5591.

Additionally, about 36.6% reduction of biofilm formation was reported with 2.5% (v/v) of *V. alginolyticus* extract without significant effect on cell growth. We speculated that the reduction in biofilm formation by *V. alginolyticus* extract was associated with the loss of its ability to motility, attach to surfaces, and subsequently to form a biofilm of VP_AHPND_. For better understanding, the effect of *V. alginolyticus* extract (2.5%, v/v) was examined using SEM. The structural change in the VP_AHPND_biofilm was observed after 24 and 48 h. From the transcriptome data, we found that the genes encode fimbrial and pilus protein were down-regulated. Also, hypothetical proteins were differentially expressed by directly or indirectly. It might be play the role of biofilm formation. In the present study, we demonstrated the hypothetical model for explaining transcriptional regulation of QS in VP_AHPND_ by *V. alginolyticus* extract based on the differential gene expression with available literature (Fig. 9). In general, *V. parahaemolyticus* produces harveyi autoinducer 1 (HAI-1) produced by LuxM and recognized by histidine kinase LuxN, autoinducer 2 (AI-2) is synthesized by LuxS and detected by histidine kinase complex LuxPQ, and cholerae autoinducer 1 (CAI-1) which is synthesized by CqsA and detected by histidine kinase CqsS. When the levels of autoinducers are high and bound to receptors lead to dephosphorylation and inactivation of the LuxO repressor. The Qrr genes are not transcribed, resulting in an expressed LuxR or OpaR regulator, whereas in turn repressed the transcription of regulator AphA (Gode-Potratz & McCarter, 2011; Zhang et al., 2012). Interestingly, transcriptome analysis revealed that the expression of LuxR transcriptional regulator or OpaR homolog (TetR family transcriptional regulator), sensor histidine kinase, sigma-54 interacting response regulator (alternative sigma factor, RpoN), C-factor and capsular polysaccharide were found to be up-regulated. Generally, QS system can be blocked by stopping of signal generator (LuxI homologue), destroying the signal molecule (autoinducer), preventing or competition signal molecule from binding with to signal receptor (LuxR homologue), and interruption with folding of some protein associated with QS such as transcriptional regulator (*Rasmussen and Givskov, 2006*). Hence, it perhaps suggests that the anti-QS mechanism of a marine bacterium, *V. alginolyticus* extract could interrupt with histidine kinase receptor and/or folding protein of LuxR and RpoN transcriptional regulator involve QS-controlled phenotypes including swimming, biofilm formation, and capsular polysaccharide. However, there was no evidence on how *V. alginolyticus* extract and LuxR transcriptional regulator interact in controlling VP_AHPND_. Moreover, genes encoding for cell division, lipoprotein, chemotaxis, biotin synthase, protease, hemolysin secretion, phage shock protein, iron-regulated protein, bacterioferritin and ABC transporter were down-regulated in the presence of *V. alginolyticus* extract. A previously study reported that motility, biofilm formation, type III and VI secretion system production (T3SS, T6SS), and capsule production of *V. parahaemolyticus* are directly controlled by the QS regulator OpaR (Gode-Potratz & McCarter, 2011; Kernell Burke et al., 2015; Salomon, Klimko & Orth, 2014; Wang et al., 2013; Wu et al., 2019; Zhang et al., 2012). OpaR has been known as a regulator for the expression of various virulence factors and represses cytotoxicity toward host cells (Gode-Potratz & McCarter, 2011). Studies have demonstrated that *rpoN* mutant leads to loss of motility and also reduced biofilm formation in *V. parahaemolyticus*, *V. cholerae*, *V. fischeri*, *V. anguillarum*, and *P. aeruginosa* (Klose & Mekalanos, 1998; O’Toole et al., 1997; Whitaker, Richards & Boyd, 2013; Wolfe et al., 2004). Moreover, the deletion of *aphA* leads to reduce hemolytic and cytotoxic activity in *V. parahaemolyticus* (Wang et al., 2013). Also, the results of this study showed that hemolysin secretion which is an important virulence factor required for pathogenesis in *V. parahaemolyticus* was down-regulated in the presence of *V. alginolyticus* extract. C-factor is an intercellular signaling protein, which is required for aggregation, sporulation, and associated with the cell surface and biofilm formation (Andersen & Kaiser, 1996). Similar research revealed that the production of capsular polysaccharide was conversely related to biofilm in *Pasteurella multocida*. It is possible that a negatively charged capsule may interrupt the biofilm formation by preventing the EPS matrix from encasing bacterial cell or blocking adherence to surface (Petruzzi et al., 2017). The major compound presented in *V. alginolyticus* extract was demonstrated some resemblance with Cyclo-(L-Leu-L-Pro) and Cyclo-(L-Phe-L-Pro). Among the studies of marine isolates, the antibiotic agent pyrrolo[1,2-a]pyrazine-1,4-dione, hexahydro from *Bacillus tequilensis* strain MSI45 recovered from a marine sponge, *Callyspongia diffusa* could effectively inhibited the multi-drug resistant *Staphylococcus aureus* and showed high antioxidant activity (Kiran et al., 2018). Moreover, pyrrolo [1,2-a]pyrazine-1,4-dione, hexahydro- from *Streptomyces mangrovisoli* isolated from mangrove soil in the state of Pahang, Peninsular Malaysia exhibited strong antioxidant activity (Ser et al., 2015). In addition, Cyclo-(L-Phe-L-Pro) or pyrrolo [1,2-a]pyrazine-1,4-dione, hexahydro-3-(phenylmethyl) from *Streptomyces* sp. strain VITPK9 isolated from the salt spring habitat of Manipur, India was suggested as a potential anticandidal compound (Sanjenbam & Kannabiran, 2016). Findings from the present study revealed that four extract fractions of *V. alginolyticus* (no. 1, 4, 5, and 7) could have influenced QS system of *C. violaceum* at concentrations 1.6, 0.6, 0.4, and 0.8 mg/mL, respectively (Fig. S1). Similar research reported that 0.039 mg/mL (160 µM) Cyclo-(L-Phe-L-Pro) and 0.023 mg/mL (94 µM) Cyclo-(L-isoLeu-L-Pro) produced by *Marinobacter* sp. SK-3 inhibited QS-dependent production of violacein by *C. violaceum* CV017 and also reduced luminescence production of reporter *E. coli* pSB401 (Abed et al., 2013). Moreover, QS inhibition were achieved with 0.250 mg/mL of Cyclo-(L-Leu-L-Pro), secreted by *Staphylococcus saprophyticus* which isolated from marine sponges, and based on the inhibition of violacein production the inhibition of QS-controlled violacein production was observed with 0.250 mg/mL of Cyclo-(L-Leu-L-Pro), which secreted by Staphylococcus saprophyticus (Li et al., 2013). Hence, it perhaps suggests that Cyclo-(L-Leu-L-Pro) and Cyclo-(L-Phe-L-Pro) can be blocked using interference or competitive QS inhibition that outcompete autoinducer of VP_AHPND_ for receptors resulting in LuxR or OpaR transcriptional regulator turns on which could modulate the expression of QS-controlled phenotypes including swimming, biofilm formation, EPSs production, and capsular polysaccharide. Bacteria in the genus *Vibrio* have been successfully used as probiotic in shrimp aquaculture (Garriques & Arevalo, 1995; Griffith, 1995). This genus is found as gut microbiota of shrimp which are associated to compete with pathogenic bacteria and to produce essential elements for host metabolism (Tzuc et al., 2014). In this study, treatment with *V. alginolyticus* resulted in 80% shrimp larvae survival, whereas shrimp larvae challenged with VP_AHPND_ alone reached 55.56% mortality. Previously, a study reported that the probiotic *V. alginolyticus* strain Ili caused 20% increase in shrimp larvae survival and shrimp yield increased significantly 208 kg/ha (Rodriguez et al., 2011). Moreover, in other vibrios tested, the probiotic *V. gazogenes* has potential for the control of pathogenic bacteria in the Pacific white shrimp *L. vannamei* (Thompson et al., 2010). Additionally, in our experiments previously, isolation and characterization of *V. alginolyticus* were investigated. *V. alginolyticus* is isolated from seafood samples, Songkhla province, Thailand. The results revealed that 35 isolates of *V. alginolyticus* out of 50 isolates were identified and tested against VP_AHPND_ by cross-streak method. A total of 5 from 35 *V. alginolyticus* isolates showed antagonistic activity to VP_AHPND_ especially strain PSU5591. Interestingly, *V. alginolyticus* strain BC25 was high potential isolate. Therefore, we focus on *V. alginolyticus* BC25 against VP_AHPND_ PSU5591 for further experiments. The data presented in “The 4th National Conference on Science and Technology of Southern Network 2019 (NSCIC 2019), Songkhla Rajabhat University”. This study demonstrates the possibility of using *V. alginolyticus* as a potential source for the production of anti-QS compounds or used as probiotics in aquaculture.

## Conclusions

A marine *V. alginolyticus* BC25 exhibited anti-QS and anti-biofilm activity of VP_AHPND_ PSU5591. Based on the results of RNA-seq, bacterial extract interfered with the expression of flagella genes involved in biofilm formation and iron-regulated virulence regulatory gene of VP_AHPND_. In addition, LuxR family transcriptional regulator gene, c factor cell–cell signaling gene, and capsular polysaccharide were up-regulated. The compounds Cyclo-(L-Leu-L-Pro) and Cyclo-(L-Phe-L-Pro) were identified as a potential anti-QS compound. Also, *V. alginolyticus* has potential for the use as a probiotic in shrimp against VP_AHPND_. This study represents the attractive target for alternative strategies to control VP_AHPND_ in aquaculture.

##  Supplemental Information

10.7717/peerj.11567/supp-1 Supplemental Information 1 Analysis of active fractions of *Vibrio alginolyticus* BC25 showing anti-QS activity HPLC analysis of *Vibrio alginolyticus* BC25 extract. The chromatogram shows the seven main active peaks. Analysis was performed on a reverse-phase C18 column (50 × 2.1 mm, Waters, CA, USA). The mobile phase was methanol and water (20:80, v/v) at 30° C at a flow rate of 0.2 mL/min. The insert picture in (Fig. S1) is the re-tested of the seven peak compounds at concentrations ranging from 0.4−1.6 mg/mL against violacein production in *C. violaceum* DMST46846 by drop plate method.

10.7717/peerj.11567/supp-2 Supplemental Information 2 Representative NMR spectroscopy analysis of *Vibrio alginolyticus* BC25 extract NMR spectrum analysis of active compound (Fraction 1). (A) ^1^H NMR spectrum at 500 MHz (B) ^13^C NMR spectrum at 125 MHz (C) 2D-HMBC spectrum (D) 2D-HSQC spectrum.

10.7717/peerj.11567/supp-3 Supplemental Information 3 List of the differential gene expression analysis (DEGs) between control and treat with *V. alginolyticus* extract

10.7717/peerj.11567/supp-4 Supplemental Information 4 Raw data obtained from the inhibition of violacein production in *C. violaceum* DMST46846 (Fig. 1A)

10.7717/peerj.11567/supp-5 Supplemental Information 5 Raw data obtained from the inhibition of biofilm formation in VP_AHPND_ PSU5591 by *V. alginolyticus* BC25 extract (Fig. 4)

10.7717/peerj.11567/supp-6 Supplemental Information 6 Raw data obtained from swimming and swarming zone diameter of VP_AHPND_ PSU5591 in the presence of CFCS of *V. alginolyticus* BC25 (Table 1)

10.7717/peerj.11567/supp-7 Supplemental Information 7 Raw data obtained from swimming and swarming zone diameter of VP_AHPND_ PSU5591 in the presence of of *V. alginolyticus* extract (Table 2)

10.7717/peerj.11567/supp-8 Supplemental Information 8 GC-MS chromatograms of *Vibrio alginolyticus* BC25 extract

10.7717/peerj.11567/supp-9 Supplemental Information 9 RNA sequencing data

## Additional Information and Declarations

### DNA Deposition

The following information was supplied regarding the deposition of DNA sequences: The RNA sequences described are available at GenBank: SRR12398138 and SRR12459902.

### Data Availability

The following information was supplied regarding data availability: The raw data are available in the Supplemental Files. *V. alginolyticus* BC25 are deposited in Thailand Bioresource Research Center (TBRC), Pathum Thanni, Thailand. Accession number: TBRC 12771.
