# Supplementary figures and images for: Vibrio alginolyticus influences quorum sensing-controlled phenotypes of acute hepatopancreatic necrosis disease-causing Vibrio parahaemolyticus
Source: PeerJ. 2021 Jun 1;9:e11567. doi: 10.7717/peerj.11567 (PMC8176930; doi:10.7717/peerj.11567)

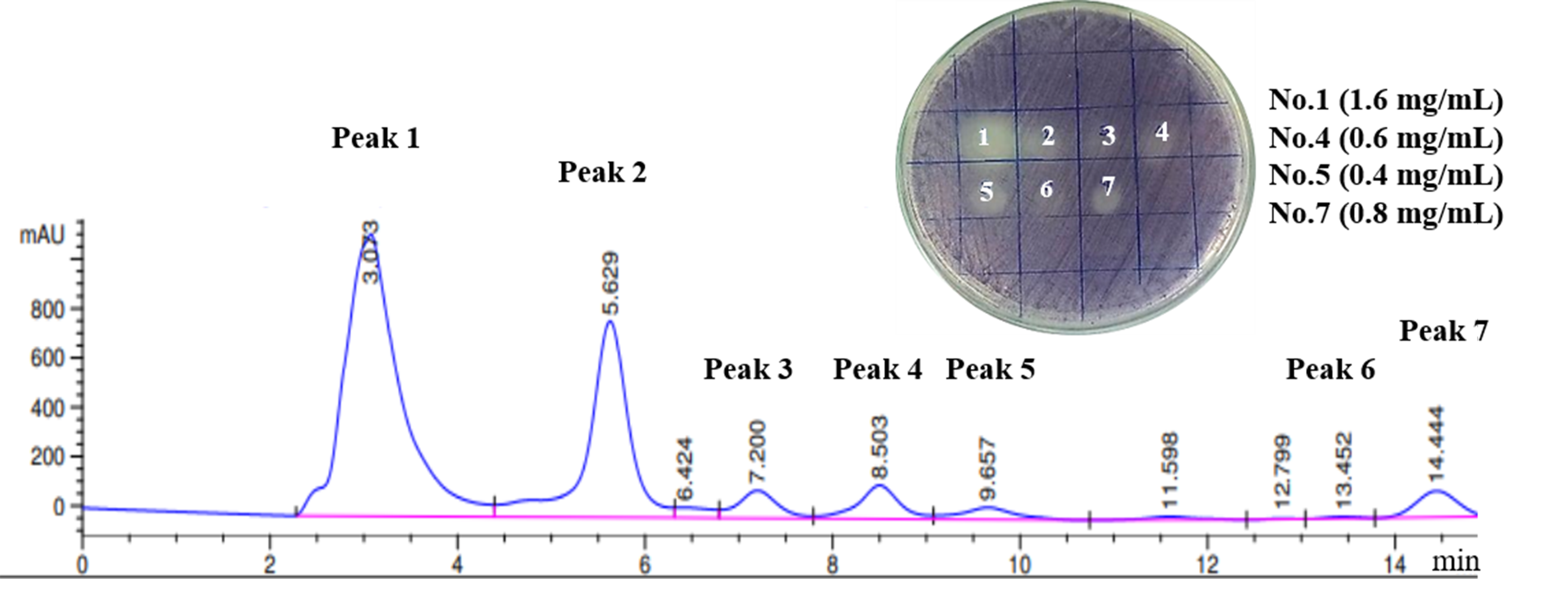

Supplement: Supplemental Information 1 — HPLC analysis of Vibrio alginolyticus BC25 extract. The chromatogram shows the seven main active peaks. Analysis was performed on a reverse-phase C18 column (50 × 2.1 mm, Waters, CA, USA). The mobile phase was methanol and water (20:80, v/v) at 30° C at a flow rate of 0.2 mL/min. The insert picture in (Fig. S1) is the re-tested of the seven peak compounds at concentrations ranging from 0.4−1.6 mg/mL against violacein production in C. violaceum DMST46846 by drop plate method. [file peerj-09-11567-s001.png]

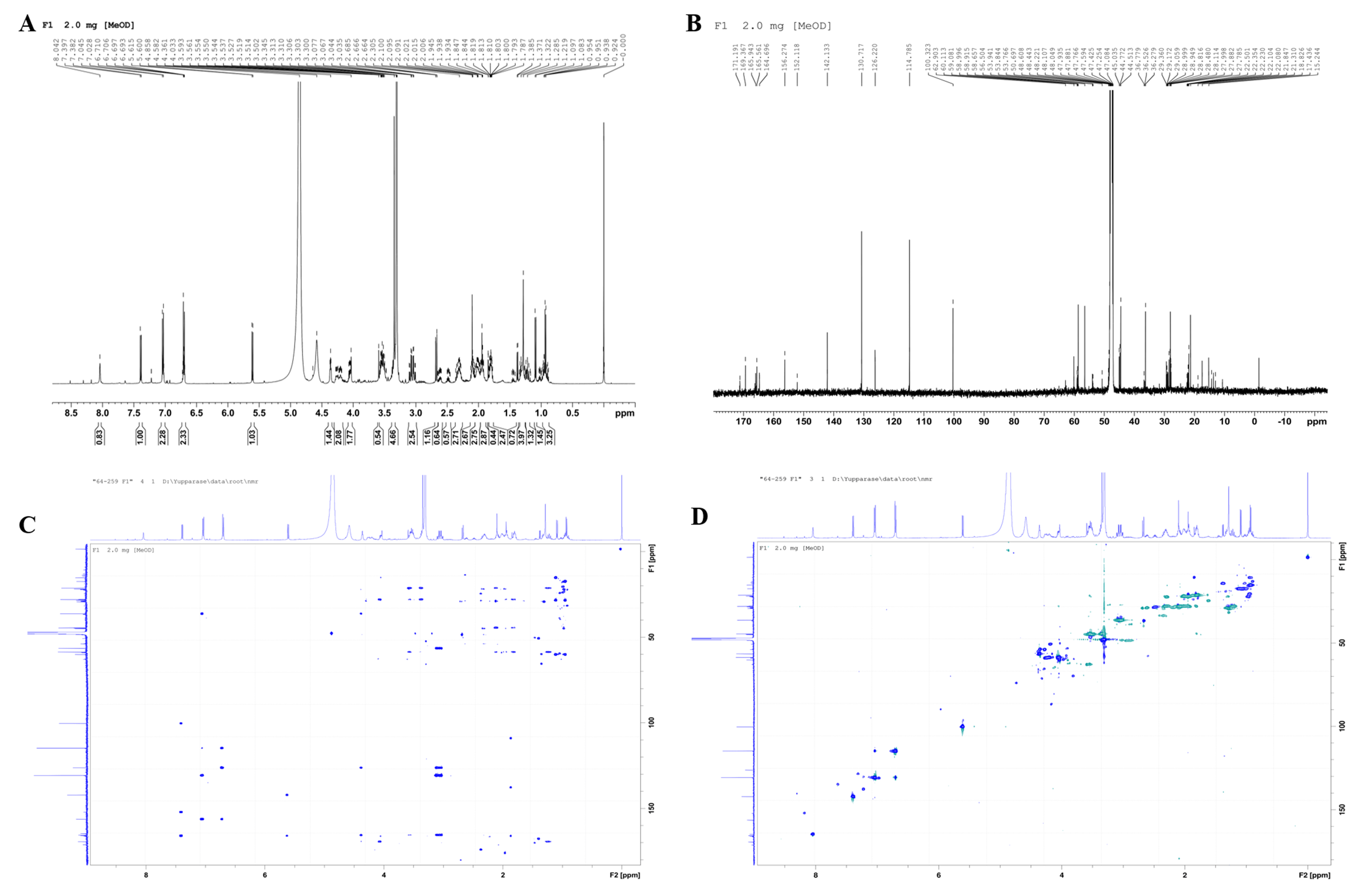

Supplement: Supplemental Information 2 — NMR spectrum analysis of active compound (Fraction 1). (A) 1H NMR spectrum at 500 MHz (B) 13C NMR spectrum at 125 MHz (C) 2D-HMBC spectrum (D) 2D-HSQC spectrum. [file peerj-09-11567-s002.png]
